# Prevalence of Mental Health Problems in Transgender Children Aged 9 to 10 Years in the US, 2018

**DOI:** 10.1001/jamanetworkopen.2022.23389

**Published:** 2022-07-22

**Authors:** Douglas H. Russell, Monsurul Hoq, David Coghill, Kenneth C. Pang

**Affiliations:** 1 Murdoch Children’s Research Institute, Parkville, Victoria, Australia; 2 Department of Paediatrics, University of Melbourne, Victoria, Australia; 3 Department of Psychiatry, University of Melbourne, Victoria, Australia; 4 Royal Children’s Hospital, Melbourne, Victoria, Australia

## Abstract

This cohort study evaluates the prevalence of mental health problems in transgender and gender diverse children aged 9 to 10 years in the US.

## Introduction

Research into the mental health of transgender and gender diverse (henceforth, *transgender*) young people points to concerning levels of depression, anxiety, and other mental health issues.^1,2,3,4,5^ Much of this research has focused on transgender young people attending specialist gender clinics^1,3,4,5^ or recruited via community-based convenience sampling.^2^ Although such studies have made important contributions to understanding the mental health of transgender young people, they have critical limitations and potential biases. Clinical samples may misestimate the prevalence and severity of mental health problems, while convenience samples may experience selection bias. Both cases lead to challenges in generalizing findings to the general population. To address these shortcomings, research using representative community samples of transgender young people is required.

## Methods

This cohort study compared mental health outcomes between transgender and cisgender children aged 9 to 10 years who completed baseline assessment in the Adolescent Brain Cognitive Development study, which recruited more than 11 000 children across the US using multistage probability sampling with the aim of obtaining a representative sample of the US population. The Adolescent Brain Cognitive Development study was approved by the institutional review board at the University of California, San Diego. Parents provided informed consent and children additionally gave their assent. Using the parent-reported Child Behavior Checklist, we assessed standardized T scores for 6 *Diagnostic and Statistical Manual of Mental Disorders, Fifth Edition* (*DSM-5*)–oriented subscales (depressive/anxiety/somatic/attention-deficit/hyperactivity disorder/oppositional defiant/conduct problems) and suicidality. Data were analyzed using Stata statistical software version 17 (StataCorp). We calculated the odds ratio using multilevel logistic regression. We did not set any arbitrary cut off defining a priori level of significance based on best practice. We have used odds ratios to report the effect size and confidence interval to report the variability of the estimate in the general population. Data were analyzed in March 2022. This study followed the Strengthening the Reporting of Observational Studies in Epidemiology (STROBE) reporting guideline. Additional methods are outlined in the eMethods in the Supplement.

## Results

This analysis included 7169 children and compared transgender (58 participants) and cisgender (7111 participants) children who understood and answered the question “Are you transgender?” The 4692 participants who reported not understanding this question were excluded along with 17 who did not record a response. The mean (SD) age of participants was 10.03 (0.62) years. Transgender children represented 0.8% (weighted) of respondents and for all 6 subscales recorded higher mean T scores, although these were all in the reference range and the standardized mean difference in each case was small (Table). We also determined the proportion of cisgender and transgender children who scored in the borderline or clinical range (T ≥65) for each subscale (Table). The odds ratio of transgender children being in this range was increased for all 6 subscales (range, 1.57 [95% CI, 0.50-4.91] to 3.13 [95% CI, 1.46-6.71]) as well as for suicidality (odds ratio, 5.79 [95% CI, 2.08-16.16]), although the results for attention-deficit/hyperactivity disorder and oppositional defiant problems were not statistically significant (Table).

**Table.** A Comparison of Transgender and Cisgender Participants According to 6 *DSM-5*–Oriented Subscales and Suicidality Questions from the CBCL

*DSM-5 *oriented subscales	T score on CBCL subscale			Proportion scoring above clinical cutoff		
	Mean (SE)^a^		Difference in mean (95% CI)^a^	Participants, No. (%)^a^		OR (95% CI)^a^^,^^b^
	Cisgender (n = 7111)	Transgender (n = 58)		Cisgender (n = 7111)	Transgender (n = 58)	
Depressive problems	53.7 (0.08)	57.3 (1.27)	−3.61 (−3.89 to −3.33)	491 (7.8)	8 (18.1)	2.53 (2.53 to 2.53)
Anxiety problems	53.4 (0.08)	57.0 (1.25)	−3.58 (−3.86 to −3.30)	527 (8.0)	10 (18.7)	2.70 (1.43 to 5.11)
Somatic problems	55.2 (0.09)	57.0 (0.97)	−1.79 (−2.05 to −1.53)	884 (13.4)	12 (20.3)	1.62 (1.62 to 1.62)
Attention-deficit/hyperactivity disorder	53.2 (0.08)	55.6 (1.31)	−2.36 (−2.65 to −2.07)	431 (6.4)	5 (10.2)	1.57 (0.50 to 4.91)
Oppositional defiant problems	53.6 (0.07)	55.8 (1.51)	−2.21 (−2.51 to −1.90)	415 (6.1)	5 (13.6)	2.39 (0.85 to 6.77)
Conduct problems	53.2 (0.08)	56.4 (1.52)	−3.2 (−3.52 to −2.88)	472 (7.1)	9 (18.4)	3.13 (1.46 to 6.71)
Suicidality^c^	NA	NA	NA	202 (2.9)	9 (14.8)	5.79 (2.08 to 16.16)

Abbreviations: CBCL, Child Behavior Checklist; *DSM-5*, *Diagnostic and Statistical Manual of Mental Disorders, Fifth Edition*; NA, not applicable; OR, odds ratio.

^a^ All estimates are weighted using the population weights from the American Community Survey and controlling for the multilevel clusters.

^b^ ORs were calculated using multilevel mixed-effect logistic regression.

^c^ Measured by summing the 2 suicide-related items in the parent-report CBCL which assess suicidal ideation and attempts.

## Discussion

Previous research using clinical samples of transgender children aged 5 to 11 years^4^ reported lower rates of depression and anxiety than we observed in this cohort study. Apart from methodological differences in assessing mental health, a possible reason for this disparity is that transgender children attending specialist gender clinics are likely to have support from their families (a key protective factor for the mental health of transgender young people); in comparison, many transgender children in the general population lack parental support for their gender.

Previous studies using clinical and convenience samples of transgender adolescents had higher rates of depression and anxiety compared with our sample.^1,2,3,5^ This is consistent with earlier clinic-based observations that transgender children have lower rates of anxiety and depression compared with transgender adolescents,^4^ which may be explained by observations from the general population that depression and anxiety more frequently develop during adolescence.^6^

The small number of transgender participants is a limitation of our study, as is the exclusion of many children who did not understand the question on gender identity. Nevertheless, this is, to our knowledge, the first study to report rates of *DSM-5*–related problems using a representative population sample of transgender children. Our findings suggest that by 9 to 10 years of age transgender children already show increased susceptibility to mental health problems compared with their cisgender peers, which has important public health implications. Whether this is due to stigma, minority stress, discrimination, or gender dysphoria is unclear, but providing appropriate mental health supports to this vulnerable group is paramount.

## Notes

Supplement.
eMethods.

eReferences
