# Supplementary material for: Prevalence of Mental Health Problems in Transgender Children Aged 9 to 10 Years in the US, 2018
Source: JAMA Netw Open. 2022 Jul 22;5(7):e2223389. doi: 10.1001/jamanetworkopen.2022.23389 (PMC9308051; doi:10.1001/jamanetworkopen.2022.23389)
Supplement: Supplement. — eMethods. eReferences [file jamanetwopen-e2223389-s001.pdf]

Supplemental Online Content

Russell DH, Hoq M, Coghill D, Pang KC. Prevalence of mental health problems in transgender children aged 9 to 10 years in the US, 2018. *JAMA Netw Open*. 2022;5(7):e2223389. doi:10.1001/jamanetworkopen.2022.23389

eMethods.

eReferences

This supplemental material has been provided by the authors to give readers additional information about their work.

© 2022 Russell DH et al. *JAMA Network Open.*

eMethods

Baseline ABCD data were collected from 2016 with plans for follow up over a period of ten years. Demographic information regarding the participants at baseline can be found in Heeringa et al. 2020[1]. Our analysis of the data was done in 2021 using Stata, version 17[2] (Stata Corp, 2021).

The ABCD study relies on a central Institutional Review Board (cIRB) at the University of California, San Diego for the ethical review and approval of the research protocol[3]. This approval included the use of the informed consent procedure as outlined in Clark et al. 2018[4] in which parents gave informed consent and participants aged 9-10 years additionally gave assent.

Statistical Analysis

We calculated the odds ratio using multilevel logistic regression. We did not set any arbitrary cut off defining a priori level of significance based on best practice[5]. We have used odds ratio to report the effect size and confidence interval to report the variability of the estimate in the general population.

eReferences

1. Heeringa SG, Berglund, PA. A guide for population-based analysis of the Adolescent Brain Cognitive Development (ABCD) Study baseline data. [published online February 10, 2020]. *BioRxiv.* doi.org/10.1101/2020.02.10.942011
2. StataCorp. 2021. *Stata Statistical Software: Release 17*. College Station, TX: StataCorp LLC.
3. Auchter AM, Mejia MH, Heyser CJ, et al. A description of the ABCD organizational structure and communication framework. *Dev Cog Neurosci.* 2018;32:8-15.
4. Clark DB, Fisher CB, Bookheimer S, et al. Biomedical ethics and clinical oversight in multisite observational neuroimaging studies with children and adolescents: The ABCD experience. *Dev Cog Neurosci*. 2018;32:143-154.
5. Amrhein V, Greenland S, McShane B. Scientists rise up against statistical significance. *Nature.* 2019;567:305-307

© 2022 Russell DH et al. *JAMA Network Open.*
